# 

**Published:** 1900-01

**Authors:** 


### LIST OF CONTRIBUTORS TO VOLUME XXI.

Allan, Dr. C. F.
Allan, Dr. Geo. S.
Arrington, Dr. B. F.
Barker, Dr. C. C.
Barton, Walter W., D.D.S.
Boardman, Waldo E., D.M.D.
Bogue, E. A., M.D.
Bogue, Dr. Frederic L.
Briggs, Dr. Edward C.
Clapp, Dwight M., D.M.D.
Daly, James H., D.D.S.
Davis, Edwin E., A.B., D.D.S.
Dickerman, Frank R., D.M.D.
Eliot, Charles W., L.L.D.
Evans, W. Warrington, M.D., D.D.S.
Fellows, A. P., D.D.S.
Flanagan, Dr. A. J.
Fillebrown, Thomas, M.D., D.M.D.
Freeman, Dr. S.
Gooding, R. L., D.D.S.
Gordon, Rev. Dr. George A.
Grant, Harry L., D.M.D.
Howe, J. Morgan.
Johnson, C. N., L.D.S., D.D.S.
Kimball, Charles Otis, A.B., M.D.
Kelley, H. A., D.M.D.
Learned, Dr. J. B.
Locherty, Dr. James B.
Luckie, S. B., D.D.S.
Martinez, Julio R., D.D.S.
McClain, John A., D.D.S.
Meriam, Horatio C.
Mills, Dr. G. Alden.
Minot, Dr. C. S.
Neall, Walter H., D.D.S.
Palmer, James G.
Palmer, Dr. S. B.
Parker, Dr. C. B.
Peirce, C. N., D.D.S.
Porter, Charles A., M.D.
Potter, Wm. H., D.M.D.
Raymond, E. H., D.D.S.
Rollins, Dr. William.
Smith, B. Holly, M.D.
Smith, D. D., D.D.S., M.D.
Talbot, Eugene S., M.D., D.D.S.
Taylor, Dr. Levi C.
Thompson, A. H., D.D.S.
Trueman, William H., D.D.S.
Whitney, Cephas, D.D.S.
Williams, Harold, M.D.
Zerfing, Wilson, D.D.S.

